# Editorial: The chemistry of food in the advent of sustainable diets

**DOI:** 10.3389/fnut.2022.1077985

**Published:** 2022-11-07

**Authors:** Márcio Carocho, Lillian Barros, Patricia Morales, Spyridon A. Petropoulos, Marina Soković

**Affiliations:** ^1^Centro de Investigação de Montanha (CIMO), Instituto Politécnico de Bragança, Bragança, Portugal; ^2^Dpto. Nutrición y Ciencia de los Alimentos, Facultad de Farmacia, Universidad Complutense de Madrid (UCM), Madrid, Spain; ^3^Department of Agriculture Crop Production and Rural Environment, University of Thessaly, Volos, Greece; ^4^Institute for Biological Research “Siniša Stanković”-National Institute of Republic of Serbia, University of Belgrade, Belgrade, Serbia

**Keywords:** diet, sustainability, chemistry, food, circularity

Climate change and the global population increase puts food security and food safety under threat. Moreover, the unsustainability of the current dietary habits needs urgent attention and more sustainable patterns have to be adopted to allow the access to food sources for all. In this context, scientific innovations in chemistry, microscopy, analytical methodology and many other food science-related disciplines are pivotal for the design of new healthy foods that are produced under sustainable and environment friendly conditions, while they ensure food safety.

The Research Topic “The chemistry of food in the advent of sustainable diets” focused on how research on food could help shape future diets, making them more sustainable and in line with the circular and green economy. The seven accepted manuscripts touch upon these concerns from various standpoints and help shape the path for the advent of these sustainable diets.

Husain et al. focused their research on a purple pigment, delphinidin, which could take two forms, namely the anthocyanin and anthocyanidin. They reviewed the chemistry, biosynthesis, stability and physicochemical parameters of this natural pigment. The Research Topic also includes three research articles focused on sustainable crops and how they could be improved. In particular, Dai et al. studied the stalks of *Rheum officinale* and *Rheum tanguticum* for their nutritional properties, bioactive compounds and anti-inflammatory activity, concluding that they could be used as alterntive crops with interest for the industry. Beyond the Earth's diet, Duri et al. studied the influence of simulated Martian and Lunar soils with varying amounts of animal manure on lettuce, finding that the use of manure could improve certain phenolic and bioactive properties, making this valuable leafy crop very important for the sustainment of future space colonies. Aspleniaceae ferns and *Ceterach officinarum* were studied by Farràs et al., who determined their polyphenol profile as well as the antioxidant activity in cell lines, concluding that these species do have cytoprotective effects. In the work of Xu et al., largemouth bass and the influence on its growth, flesh quality and metabolomics by comparing formula vs. trash fish was evaluated. The authors concluded that formula feeding improved the fatty acid profile as well as flesh texture although the free amino acids content was reduced. Finally, Donini et al. reported nutritional indicators to assess the sustainability of a healthy diet. This article summed up the spirit of the Research Topic, although focusing on the Mediterranean diet, by pointing out that nutrition indicators will be useful to design new diets but also preserve cultural heritage of sustainable traditional diets.

Overall, the issue includes seven articles which contribute to new and updated knowledge on the chemistry of foods and how they can be incorporated in newly and sustainably designed diets. The published articles are just a fraction of the research that can be focused on new diets. Beyond being urgent, the sustainability of future diets is essential from a societal point of view since it has to guarantee that everyone around the globe gets secure and safe food everyday.

## Author contributions

All authors listed have made a substantial, direct, and intellectual contribution to the work and approved it for publication.

## Conflict of interest

The authors declare that the research was conducted in the absence of any commercial or financial relationships that could be construed as a potential conflict of interest.

## Publisher's note

All claims expressed in this article are solely those of the authors and do not necessarily represent those of their affiliated organizations, or those of the publisher, the editors and the reviewers. Any product that may be evaluated in this article, or claim that may be made by its manufacturer, is not guaranteed or endorsed by the publisher.

